# From abstract to article: publication rates of abstracts presented at the Society for Healthcare Epidemiology of America spring conference 2018 and 2021

**DOI:** 10.1017/ice.2025.10348

**Published:** 2026-01

**Authors:** Aayushi Rajani, Shifa Karatela, Lipi Modha, Hitanshi Bhuptani, Purav Shah, Abhijeet Shukla, Juhi Amin, Devisha Gandhi, Rohit Chitale, Ravi Durvasula, Justin Oring

**Affiliations:** 1 Department of Internal Medicine, University of Missouri Health Care, Columbia, MO, USA; 2 Department of Medicine, Medical College Baroda and Sir Sayajirao General Hospital, Vadodara, India; 3 Department of Medicine, Pandit Deendayal Upadhyay Medical College, Rajkot, India; 4 Department of Obstetrics and Gynecology, Medical College Baroda and Sir Sayajirao General Hospital, Vadodara, India; 5 Department of Medicine, GMERS Medical College and Hospital, Gotri, Vadodara, India; 6 Division of Infectious Diseases, Mayo Clinic Florida, Jacksonville, FL, USA

## Abstract

The publication rate of abstracts presented at a conference can provide some insight into its academic quality, although it is hardly the sole metric. We evaluated 351 SHEA Spring Conference abstracts; 49.9% were published. Findings demonstrate the strong academic output of SHEA conferences.

## Background

Conferences play a pivotal role in advancing science by enabling researchers to disseminate findings, receive real-time feedback, and foster collaborations. Abstracts, though brief, are integral to this process, allowing investigators to present preliminary results and refine their work. However, conferences are not endpoints; the expectation is that abstracts evolve into full-length publications, ensuring the research contributes meaningfully to the scientific literature.^1^ Publication rates of conference abstracts serve as measurable indicators of academic quality, reflecting both research rigor and dissemination. Yet, despite their scientific value, a substantial proportion of abstracts never reach full publication. Cochrane’s systematic reviews have reported overall publication rates ranging from 37%^2^ to 45%,^3^ highlighting concerns about lost research^4^ and potential publication bias, which can distort evidence synthesis and limit knowledge translation.

## Methods

We retrospectively analyzed the publication outcomes of abstracts presented at the Society for Healthcare Epidemiology of America (SHEA) Spring Conferences 2018 and 2021. Abstracts were sourced from the official online archive of the 2018 conference and the *Antimicrobial Stewardship & Healthcare Epidemiology* (ASHE) supplement for 2021. The outcome was defined as full-length publication status, determined through a systematic search conducted up to January 10, 2025. A total of 351 abstracts were screened using a standardized manual search protocol to enhance interrater reliability. Investigators searched Google, Google Scholar, and PubMed by combining titles, author names, and keywords, reviewing the top 10 results from each database. Abstracts were considered published if they shared at least three keywords, included at least one common author, and appeared in or after the year of presentation. The most recent one-year journal impact factor available at the time of the search was used for analysis. Data were compiled in Excel, with abstracts categorized as published or not. Qualitative variables were summarized as frequencies and percentages, while quantitative variables were reported as means with standard deviations. Comparative analyses used Chi-square tests, odds ratios, and Student’s t-tests. A *p*-value < 0.05 was considered statistically significant.

## Results

**Figure 1.**
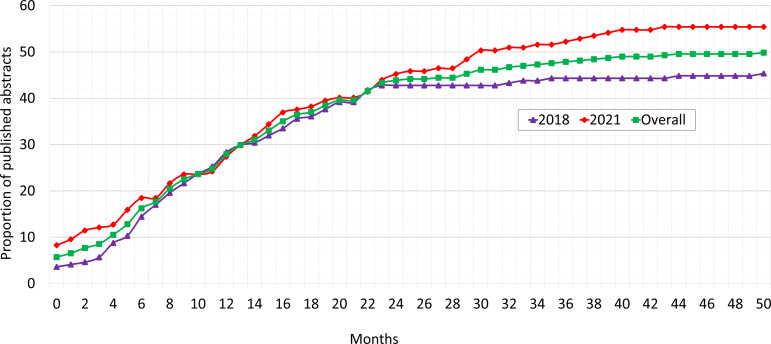
Proportion of abstracts published versus time to publication, SHEA spring 2018 and 2021 conferences.

Overall, 175/351 (49.9%) abstracts were published as full articles, 88/194 (45.4%) from 2018 and 87/157 (55.4%) from 2021. The trend in time to publication is depicted in Figure 1, which illustrates the proportion of abstracts published over time following presentation, with 17 publications occurring before the conference and recorded as having zero months to publication. The mean time to publication was 13.9 ± 10.6 months, range: 0–51. Abstracts from 2021 showed a slightly longer mean time (14.9 ± 11.7, range: 0–44) compared to 2018 (12.9 ± 9.4, range: 0–51), though the difference was not statistically significant (t(349) = –1.29, *p* = 0.19).

Table 1 presents the analytical comparison of factors associated with the publication rates of abstracts. Abstracts presented in 2021 and those delivered orally demonstrated a higher, though non-significant, likelihood of publication (*p* = 0.06 and *p* = 0.66, respectively). By study design, case series had the lowest publication rate (16.7%), whereas original articles and systematic reviews/meta-analyses (SRMAs) showed higher but non-significant rates. Abstracts with authors from different institutions had 2.54 times higher odds of publication compared with those from a single institution (*p* = 0.002). Similarly, abstracts with more than six authors demonstrated 1.95 times higher odds of publication than those with six or fewer authors (*p* = 0.003).

**Table 1.** Analytical comparison of variables among published and non-published abstracts from the SHEA spring 2018 and 2021 conferences

Variables (*n*)	Published, *n* (%)	Not published, *n* (%)	Chi-square statistics, *p*-value	Odds ratio (95% CI), *p*-value
Year of presentation				
2021 (157)	87 (55.4)	70 (44.6)	*X* ^2^ (1, *n* = 351) = 3.5, p = 0.06	1.49 (0.98–2.28), *p* = 0.06
2018 (194)	88 (45.4)	106 (54.6)		
Overall (351)*	175 (49.9)	176 (50.1)		
Type of presentation				
Oral presentation (67)	35 (52.2)	32 (47.8)	*X* ^2^ (1, *n* = 351) = 0.18, *p* = 0.66	1.12 (0.66–1.92), *p* = 0.66
Poster presentation (284)	140 (49.3)	144 (50.7)		
Article type^†^				
Case series (6)	1 (16.7)	5 (83.3)	*X* ^2^ (3, N = 351) = 0.24, *p* = 0.24	0.19 (0.02–1.69), *p* = 0.14
Original article^‡‡^ (326)	162 (49.7)	164 (50.3)		0.91 (0.40–2.05), *p* = 0.82
SRMA (2)	1 (50)	1 (50)		1.01 (0.06–16.21), *p* = 0.99
Other/not listed (17)	11 (64.7)	6 (35.3)		1.9 (0.69–5.26), *p* = 0.22
Affiliation of authors^‡^				
Different (70)^§^	42 (60)	28 (40)	*X* ^2^ (1, *n* = 194) = 9.5, *p* = 0.002**	2.54 (1.39–4.64), *p* = 0.002**
Same (124)^||^	46 (37.1)	78 (62.9)		
Number of abstract authors				
More than 6 (135)	81 (60)	54 (40)	*X* ^2^ (1, *n* = 351) = 9.02, *p* = 0.003**	1.95 (1.26–3.01), *p* = 0.003**
Less than or equal to 6 (216)	94 (43.5)	122 (56.5)		

Abbreviations: SRMA, Systematic Review and Meta-Analysis.

* Overall publications are not considered in the statistical analysis.

^†^All 4 study designs were evaluated together using the chi-squared test and individually by odds ratio.

^‡‡^Original article encompassed randomized controlled trials, case–control, cohort, cross-sectional, observational, and quasi-experimental studies.

^‡^Analysis was done only for 2018 abstracts; 2021 data were unavailable.

^§^At least one author from a different institution.

^||^All authors from the same institution.

**Statistically significant at *p* < 0.05.

The mean number of authors increased significantly from abstract to final publication (6.4 ± 3.8, range: 1–25 vs. 8.5 ± 5.2, range: 1–37; t(525) = –5.25, *p* < 0.001). The mean journal impact factor (IF) of publications was 4.56 ± 6.53. Oral presentations tended to appear in higher-IF journals compared to posters (5.99 ± 10.31, range: 0.44–63.5 vs. 4.19 ± 5.14, range: 0.44–53), though this difference was not statistically significant (t(173) = 1.47, p = 0.14). Similarly, 2018 abstracts were published in journals with higher IFs than those from 2021 (4.88 ± 6.96 vs. 4.22 ± 6.08), without statistical significance (t(173) = 0.67, *p* = 0.50).

Changes in authorship were frequent: only 34 (19.4%) publications retained the original authorship order and count. 141 (80.6%) showed changes, with 51 (29.1%) having a different first author. The most common journal of publication was Infection Control & Hospital Epidemiology (51; 29.1%), followed by the American Journal of Infection Control (34; 19.4%), Antimicrobial Stewardship & Healthcare Epidemiology (16; 9.1%), Open Forum Infectious Diseases (10; 5.7%), and Clinical Infectious Diseases (7; 4%).

## Discussion

When compared with other infectious disease conferences, SHEA’s publication rate of 49.9% was substantially higher than that reported for ID Week 2017–2018 (26.6%)^5^ and ICAAC 1999–2000 (36%).^6^ Relative to other internal medicine subspecialties, SHEA’s publication rate exceeded that of gastroenterology (ACG 2008, 31.5%)^7^ and cardiology (AHA 2006–08, 34.5%),^8^ while being slightly lower than rheumatology (ACR/ARHP 2006, 59.1%).^9^ These findings position the SHEA Spring Conference as a strong contributor to academic output and highlight its role in shaping the evidence base for infection prevention and healthcare epidemiology.

The non-publication of nearly half of the presented abstracts raises concerns of publication bias. Although our study did not assess the reasons for non-publication, prior reports suggest multiple contributing factors, including studies with null results being less favored for publication,^10^ time constraints, and competing priorities.^4^ Many abstracts are submitted by students, residents, or other trainees whose limited tenure at an institution may result in a loss of support and momentum to convert an abstract into a full publication. Non-publication of these abstracts can bias evidence-based guidelines, which rely on comprehensive published data to inform clinical decision-making, highlighting the need to maximize the dissemination of conference proceedings.

## Bias and limitations

The titles of abstracts may change slightly or even completely from the abstract to the full publication. This can make it difficult, or sometimes impossible, to accurately identify corresponding published articles, potentially leading to an underestimation of the true publication rate. Inter- and intra-rater reliability in matching abstracts to published articles was not assessed. Rosmarakis et al. employed a more rigorous approach, with two independent investigators reviewing each abstract-publication pair and conducting detailed data comparisons to ensure accuracy and enhance inter-rater reliability.^6^ This study was limited to data from a single healthcare epidemiology conference, limiting generalizability to broader global trends in abstract-to-publication conversion.
